# Description of a new species of *Stueningeria* Lehmann, 2019 (Lepidoptera, Cossoidea, Metarbelidae) from Kinmen Island of Taiwan

**DOI:** 10.3897/BDJ.13.e161543

**Published:** 2025-07-24

**Authors:** Shen-Horn Yen, Shih-Rei Liao, Manupa Pabasara Wickramasinghe, Chung-Te Cheng

**Affiliations:** 1 Department of Biological Sciences, National Sun Yat-Sen University, Kaohsiung, Taiwan Department of Biological Sciences, National Sun Yat-Sen University Kaohsiung Taiwan; 2 McGuire Center of Lepidoptera and Biodiversity, Florida Museum of Natural History, Gainesville, Florida, United States of America McGuire Center of Lepidoptera and Biodiversity, Florida Museum of Natural History Gainesville, Florida United States of America

**Keywords:** Metarbelidae, Indarbela, Stueningeria, Marcopoloia, Arbela, bark-feeder

## Abstract

**Background:**

Before the present study, the family Metarbelidae in Taiwan was known for only one species, *Marcopoloiadiscipuncta*. During an investigation of the lepidopteran fauna of Kinmen island in 2022, a *Stueningeria* species that had never been reported by any of previous entomological studies on Kinmen was discovered.

**New information:**

Based on the morphological characters distinct from other congeneric species, we determine the specimens from Kinmen as a species new to science. In addition, “*Arbeladea*" was often regarded as a timber pest in China and Taiwan. This species, however, has already been transferred to *Marcopoloia* and restricted to Myanmar. Another pest species, which has been misidentified as “*Arbelabaibarana*” (a junior synonym of *Marcolopoiadiscipuncta*) in Chinese literature for long, is actually belonging to *Stueningeria*. We therefore consider the taxonomic identity of the *Stueningeria* in southern China needs to be clarified when sufficient specimens and molecular data become available.

## Introduction

The family Metarbelidae is a small group within Cossoidea with around 250 species in 30 genera distributed across Afrotropial, Oriental and Indo-Australian Regions (Heppner 1991, Edwards et al. 1998, Heppner 1996, Heppner 1998, Heppner 2004, Lehmann 2007, Heppner et al. 2024). The phylogenetic placement of this family was mentioned by various authors, based on different sources of characters. It was retained as a family within Cossoidea by Holloway (1982), Wang (1994), Holloway et al. (2001), Regier et al. (2009), Mutanen et al. (2010), van Nieukerken et al. (2011), Heikkilä et al. (2015). It is considered to be related to either Ratardidae or Cossidae (Holloway 1986, Kobes and Ronkay 1990, Minet 1991, Owada 1993, Sugi 1995, Edwards et al. 1998), but Lehmann (2019) claimed that no synapomorphy between these families was detected.

Although some species are often mentioned as serious pests in agriculture and timber production in Southeast Asia and East Asia (Fletcher 1914, Beeson 1941, Gardner 1944, Srivastava 1962, Patel et al. 1966, Barlow 1982, Sindh and Chow 1983, Chang and Tong 1995, Mathew 1997, Srivastava 1998, Chen and Gu 2000, Wang et al. 2012, Chen et al. 2016, Raidas et al. 2016, Shi et al. 2016, Tripathy 2020, Raja Rishi and Sundararaj 2021) the taxonomic diversity of this family received very little attention until Ingo Lehmann (Lehmann 1997, Lehmann 2007, Lehmann 2008a, Lehmann 2008b, Lehmann 2010a, Lehmann 2010b, Lehmann 2011, Lehmann 2012, Lehmann 2013, Lehmann 2014, Lehmann 2019), Roman V. Yakovlev (Yakovlev and Zolotuhin 2020, Yakovlev and Zolotuhin 2021a, Yakovlev and Zolotuhin 2021b, Yakovlev and Zolotuhin 2021c, Yakovlev and Hulsbosch 2024) and John B. Heppner (Heppner 2004, Heppner 2005, Heppner et al. 2024) started to revise the family, based on various museum and private collections. In Asia, the species with economic importances were often identified as the members of *Arbela*, *Indarbela*, *Metarbela* or *Squamura* (Beeson 1941, Chien 1964, Barlow 1982, Chao 1995, Chen and Gu 2000, Sun et al. 2019). During the past 20 years, the genus-level taxonomy of Metarbelidae has largely been revised and at least 10 new genera have already been established to accommodate the species that were previously misplaced in several polyphyletic genera. Amongst the genera established for the Asian Metarbelidae species, *Stueningeria* exhibits the highest species diversity with 10 species (see Suppl. material 1 for the checklist of the species) distributed from India to Indo-China and south-western China (Lehmann 2019, Yakovlev and Zolotuhin 2021a, Yakovlev et al. 2022).

The potential synapomorphies of *Stueningeria* were proposed by Lehmann (2019), based on characters extracted from wing venation and genitalia of both sexes. In *Stueningeria*, the upper and lower parts of the discoidal cell of the forewing are nearly equal in size, while in other genera, the lower part of the discoidal cell is much smaller. Meanwhile, the distance between the base of forewing R2 and that of R3-R5 is much shorter than other genera. In male genitalia, *Stueningeria* exhibits a large and ovum-shaped juxta, while juxta in other genera can be small or absent. The sacculus of *Stueningeria* has a prominent digital process at the anterior end. In the female genitalia of *Stueningeria*, the apophyses anterioris arise from a narrow sclerotised band, which is actually the posterior part of the reduced A8.

In 2022, during a field survey of the insect fauna of Kinmen Island, an undescribed *Stueningeria* species was found. Amongst all the congeners, the species is most similar to *S.phaga* (Swinhoe 1894) from India, but still different in wing pattern and genitalia. In the present study, we intend to describe this new species and its biology and also to clarify the records of the metarbelid pest species in agricultural literature of Taiwan and China.

## Materials and methods

We started to collect and breed the metarbelid species from Kinmen since the summer of 2022. The larvae were all found boring into the twigs of trunks of the host trees. Since the larvae of Cossoidea only consume live tissue of the host tree (Chien 1964, Sun et al. 2019, Huo et al. 2020), we therefore cut off the section of twigs or trunks that were covered by the “frass tunnels” constructed by the larvae. The twigs or trunks were then carried back to the laboratory in NSYSU and “planted” with moisturised sands in pots. Some of the larvae and pupae were pulled out from the internal tunnels for specimen preservation. The adults were photographed and collected after eclosion. The adult specimens were then frozen in a fridge for future molecular analysis.

Dissection and mounting of genitalia followed the protocol suggested by Holloway (1976). Terminology of wing pattern followed Yakovlev and Zolotuhin (2021b) with slight modifications. We adopted Klots (1970) for terminology of genitalia for consistency.

The specimens of other *Stueningeria* species deposited in the collection of NHMUK (The Natural History Museum, London) and the first author’s private collection in NSYSU (National Sun Yat-Sen University) were examined. The type series of the new species are to be deposited in NMNS (National Museum of Natural Science, Taichung) and NHMUK, respectively.

## Taxon treatments

### Stueningeria kinmena

Yen, Wickramasinghe, Liao & Cheng sp. nov.

89A11E01-F8BB-5EBD-90D7-AF4CF3DBAF93

02B3A9CC-CF78-4437-8C1A-ED2C500577A0

#### Materials

- **Type status:** Holotype. **Occurrence:** individualCount: 1; sex: male; lifeStage: adult; establishmentMeans: native; disposition: in collection; occurrenceID: 6FAE088E-E8DB-5861-9E4B-B2D779E80C0C; **Taxon:** higherClassification: Insecta; Lepidoptera; Cossoidea; Metarbelidae; Stueningeria; **Location:** continent: East Asia; island: Kinmen; country: Taiwan; county: Kinmen; locality: Kinmen Agriculture Research Institute; verbatimLatitude: 24.448365; verbatimLongitude: 118.446909; **Identification:** identifiedBy: Shen-Horn Yen; dateIdentified: 09 November 2022; **Event:** samplingProtocol: reared from larva collected from litchi tree; eventDate: 6-11 May 2022; **Record Level:** type: PhysicalObject; language: en; institutionCode: National Sun Yat-Sen University (NSYSU); basisOfRecord: PinnedSpecimen
- **Type status:** Paratype. **Occurrence:** individualCount: 14; sex: 8 males, 6 females; lifeStage: adults; establishmentMeans: native; occurrenceStatus: present; disposition: in collection; occurrenceID: 6596D8FC-A24D-5D84-81D2-BE3A3AE03F81; **Location:** continent: East Asia; island: Kinmen; country: Taiwan; county: Kinmen; locality: Kinmen Agriculture Research Institute; verbatimLatitude: 24.448365; verbatimLongitude: 118.446909; **Identification:** identifiedBy: Shen-Horn Yen; dateIdentified: 9 November 2022; **Event:** samplingProtocol: reared from larva collected from litchi tree; eventDate: 6-11 May 2022; **Record Level:** type: PhysicalObject; language: en; institutionCode: National Sun Yat-Sen University (NSYSU); basisOfRecord: PinnedSpecimen

#### Description

**Figure 1.**
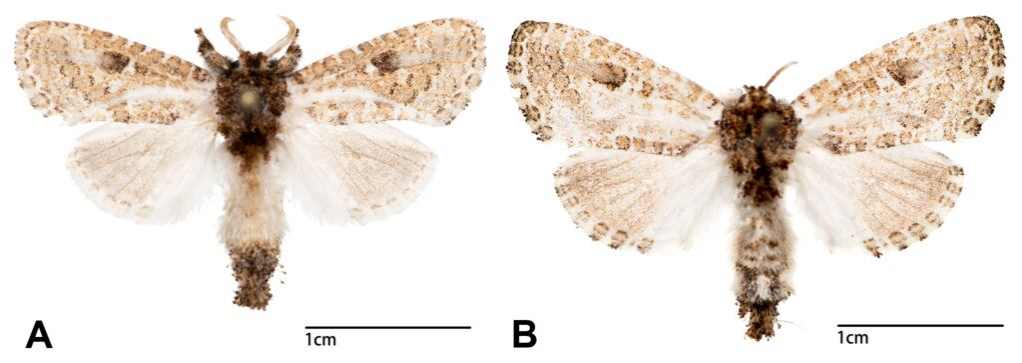
Adults of *Stueningeriakinmena* sp. nov. **A** Holotype male; **B** Paratype female.

**Wings** (Fig. 1). Length of forewing 12.5 mm on average in male (n = 9) and 13.9 mm on average in female (n = 6). Head: covered with scales light grey with brown tips; eyes light grey with black patches; labial palpus light grey. Thorax: Patagia and tegulae covered by light grey scales with brown tips. Fore-wing upperside with 12 dots between costa and Sc arranging from wing base to apex; proximal dot within discoidal cell much darker than distal one; dots on postmedial zone more conspicuous on m2, m3 and cua1 cells; dots along outer margin clearly separated by m1, m2, m3 and cua1 cells; androconial patch white, arising from wing base and extending to tornus. Forewing underside light grey, glossy, costal margin with brown dots. Hind-wing upperside creamy-white with medial to marginal zones greyish-brown; hind-wing underside with similar pattern, but lighter in colouration.

**Figure 2.**
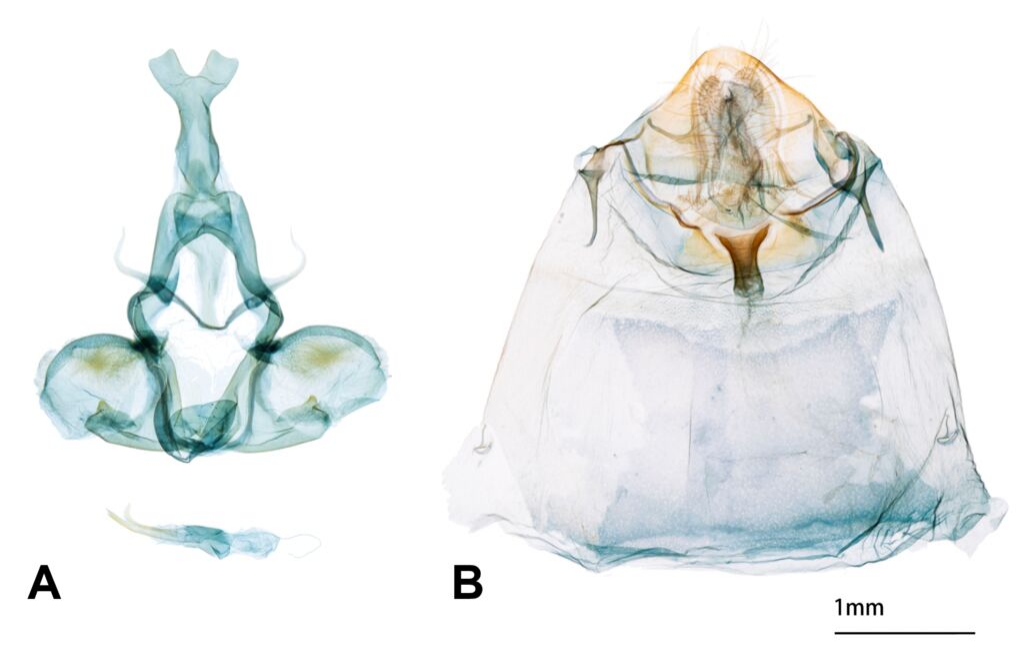
Genitalia of *Stueningeriakinmena* sp. nov. **A** Male; **B** Female.

**Male genitalia** (Fig. 2). Uncus bifurcate at posterior part; scaphium and subscaphium present and fused into a long spindle-like tube; gnathos with lateral arms lamellar and slender lateral processes upcurved; valva short and rounded with costal margin curved; sacculus much more sclerotised than other parts of valva with a triangular projection situated dorsally; juxta present and lamellar; saccus reduced; aedeagus thick with a large cornutus.

**Female genitalia** (Fig. 2). Papillae anales conical, semi-circular at apex, apophyses anteriores and posteriors short, apophyses anteriores 1.2 times longer than apophyses posteriors, antrum sclerotised, narrow, funnel-like, ductus long, corpus bursae short and small, sternite of 7^th^ abdominal segment concave at posterior and lateral margins.

#### Diagnosis

The wing pattern of *S.kinmena* sp. nov. can be distinguished from the congeners by the following characters. Compared with *S.nepalensis* (see Lehmann (2019)), colouration of the spots lying along the inner margin of fore-wing in *S.kinmena* sp. nov. are much lighter. The dark medial zone in S. campbelli (see fig. 4 in Yakovlev and Zolotuhin (2021b)) is not shown in *S.kinmena* sp. nov. Both the distal and proximal spots within the discoidal cell of fore-wing in *S.phaga* (see figs. 5-6 in Yakovlev and Zolotuhin (2021b)) are much darker than *S.kinmena* sp. nov. The postmedial zone of *S.kinmena* sp. nov. is much lighter than those of *S.htatae*, *S.csovari*, *S.ihtei* and *S.pinratanai* (see figs. 1-15 in Yakovlev and Zolotuhin (2021b)). Compared with *S.loeffleri* (see fig. 10 in Yakovlev and Zolotuhin (2021b)), the proximal dot within fore-wing discoidal cell is more rounded. The “gaps” between each spot lying on submarginal and postmedial zones of *S.kinmena* sp. nov. are clearer than those of *S.murzini*.

The male genitalia of *S.kinmena* sp. nov. resembles that of *S.phaga* (see fig. 17 in Yakovlev and Zolotuhin (2021b)), but can be distinguished by the following characters: the posterior margin of each lobe of uncus slightly concave, the outline of the postero-dorsal margin of valva more rounded and the triangular projection on valval saccus is less sharp than that of *S.phaga*.

#### Etymology

The new species is named after Kinmen Island, an island lying roughly 2 km from the south-eastern coastline of China. Kinmen is one of the few islands located close to the Chinese coastline, but under the control of Taiwan.

#### Distribution

Kinmen Island (Taiwan). This new species is very likely to be discovered in the lowlands of Fujian Province of China.

#### Ecology

**Figure 3.**
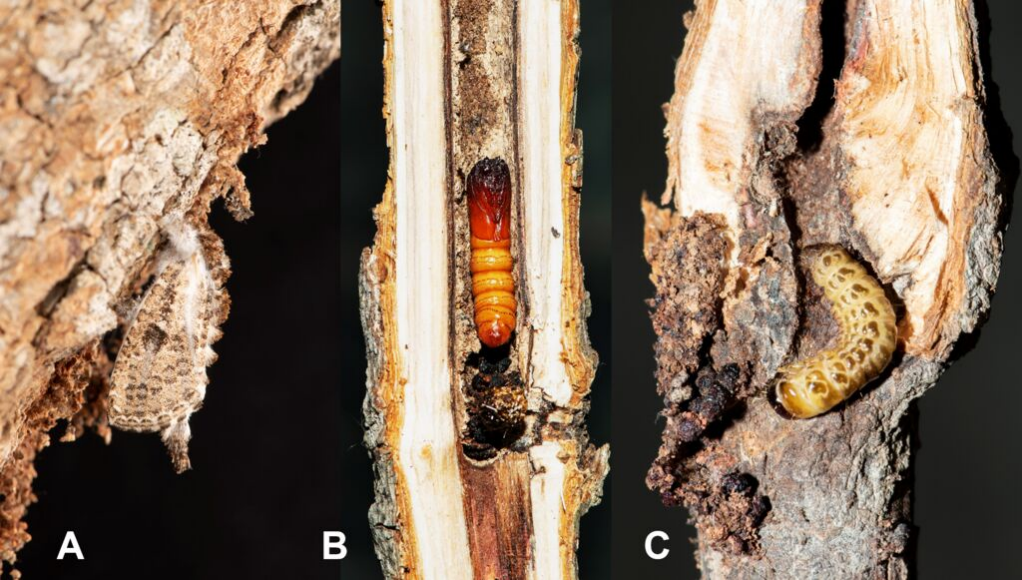
Biology of *Stueningeriakinmena* sp. nov. **A** A newly-emerged adult; **B** A pupa situated within the larval tunnel; **C** A mature larva found within the tunnel.

**Figure 4.**
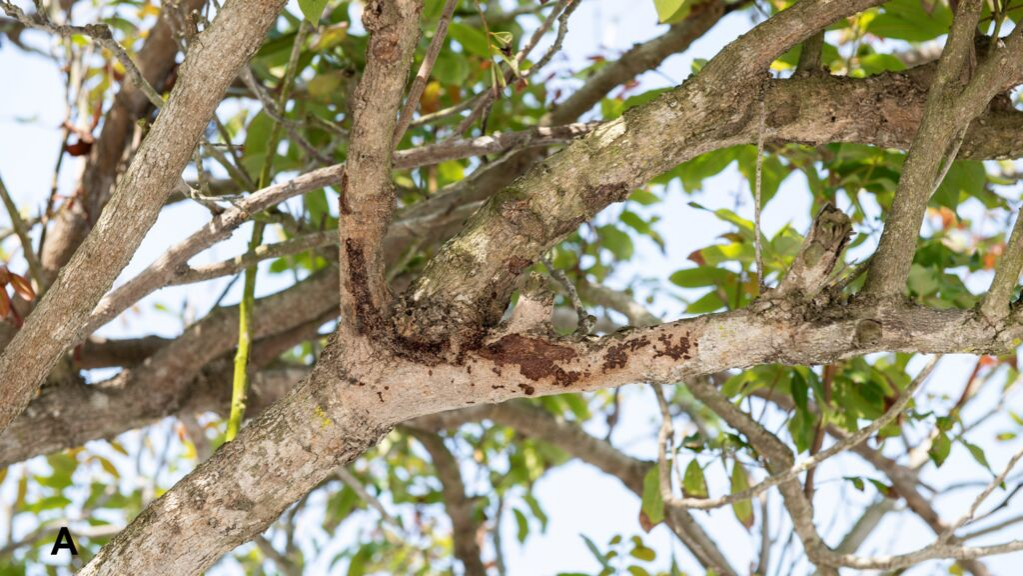
The silken tunnel made of larval frass hanging on bark of a litchi tree.

The larvae of this species are stem-borers and bark-feeders of many broad-leaved trees like the larvae of other species of Metarbelidae, but the larvae only bore into the stem when not feeding and pupation. They are mostly feeding on bark surrounding the silken tunnel mouth under a sleeve, which is made of frass and excreta of the larva (Figs 3, 4). The larvae can be found in litchi (*Litchichinensis*) and other ornamental trees (such as *Bauhiniavariegata*) in Kinmen. Adults start to emerge during May to June (Fig. 3). The female adults start to lay eggs in June and the larval stage lasts for 9 -10 months. Pupation occurs within the larval tunnel (Fig. 3). The adults can be attracted to light.

## Discussion

The investigation and inventory of the insect fauna of Kinmen have been undertaken since 1990s (Li and Ho 1990, Cheng and Yang 1998) and several publications, concentrated on selected insect groups or agricultural pests, have been published after those works (Fan et al. 2000a, Fan et al. 2000b, Huang et al. 2000, Ho and Wu 2004, Hsu 2004, Shih et al. 2004, Tung et al. 2008). Amongst all of these studies, however, Metarbelidae was neither collected nor even mentioned. We suspect that the selection of light trapping sites in those studies might be distant from Kinmen Agriculture Research Institute, where *Stueningeriakinmena* sp. nov. was first discovered. The fact that most Metarbelidae species in Taiwan and Kinmen are univoltine and having stem-boring larvae may also reduce the possibility of discovery of this family in Kinmen if the frequency of the insect survey in those studies was low.

In the agricultural and forestry literature in China and Taiwan, two metarbelid species are occasionally mentioned as serious pests of fruit trees or ornamental trees. “*Arbeladea*” was called “litchi metarbelid moth” and “*Arbelabaibarana*” was called “Acacia metarbelid moth” (Chien 1964, Chao 1995, Wang et al. 2021). Sun et al. (2019) considered the so-called “*Arbelabaibarana*” was conspecific with the so-called “*Arbeladea*” in Chinese literature. However, such confusion has been derived from a long-term ignorance of taxonomic advances in Metarbelidae in recent years. First, the real *Arbeladea* (Swinhoe 1890) had already been transferred from *Arbela* to *Marcopoloia* by Yakovlev and Zolotuhin (2021d) and, according to the specimens presented in NHMUK, this species may be restricted to Myanmar. Second, *Arbelabaibarana* is actually a junior synonym of *Marcopoloiadiscipuncta* (Wileman 1915, Inoue 1988, Yakovlev and Zolotuhin 2021c) and this species is endemic to Taiwan Island (Sonan 1938) and not present in Korea or China as suspected by Yakovlev and Zolotuhin (2021c). Finally, according to the illustration provided by Chien (1964), the so-called “*Arbelabaibarana*” is very likely a species of *Stueningeria*, but the taxonomic identity of the pest species in southern China requires careful examination of the adult specimens and analysis based on molecular data.

## Supplementary Material

XML Treatment for Stueningeria kinmena

5F7DEC5F-F23A-5816-A1D6-5C847E752F02 10.3897/BDJ.13.e161543.suppl1 Supplementary material 1 Checklist of the Species of Stueningeria Data type Taxonomic Data File: oo_1378909.xlsx https://binary.pensoft.net/file/1378909 Shen-Horn Yen, Shih-Rei Liao, Manupa Pabasara Wickramasinghe, Chung-Te Cheng
